# Prospective study of the effect of ERAS on postoperative recovery and complications in patients with gastric cancer

**DOI:** 10.20892/j.issn.2095-3941.2021.0108

**Published:** 2021-07-14

**Authors:** Ye Tian, Qiang Li, Yuan Pan

**Affiliations:** 1 Tianjin Medical University Cancer Institute and Hospital, National Clinical Research Center for Cancer, Key Laboratory of Cancer Prevention and Therapy, Tianjin, Tianjin’s Clinical Research Center for Cancer, Tianjin 300060, China

**Keywords:** Enhanced recovery after surgery, body mass index, gastric cancer, laparoscope, complication

## Abstract

**Objective:**

To study the efficacy of the enhanced recovery after surgery (ERAS) program on postoperative recovery and complications in patients with gastric cancer.

**Methods:**

Eighty patients in the perioperative period with radical gastrectomy were enrolled and randomly divided into 2 groups, the ERAS group and the non-ERAS group. The differences between the 2 groups in terms of postoperative recoveries and complications rate were determined. According to the body mass index (BMI) level, the ERAS group was divided into 2 subgroups, namely group A (BMI < 28 kg/m^2^, *n* = 16) and group B (BMI ≥ 28 kg/m^2^, *n* = 24). The non-ERAS group was also divided into group C (BMI < 28 kg/m^2^, *n* = 18) and group D (BMI ≥ 28 kg/m^2^, *n* = 22). The recovery and complications of each group were then determined.

**Results:**

The postoperative length of stay and visual analogue scale pain score were less in the ERAS group than the non-ERAS group (*P* < 0.05). Time to first postoperative exhaustion, first postoperative defecation, returning leukocyte count to normal, and stopping intravenous nutrition were significantly shorter in the ERAS group (*n* = 40), compared to the non-ERAS group (*n* = 40, all *P* < 0.05). The incidence of postoperative lower extremity intramuscular venous thrombosis was significantly higher in group D than in group B (χ^2^ = 4.800, *P* = 0.028). In addition, the incidence of lower extremity intermuscular venous thrombosis and lung infection in group D was higher than those in other groups.

**Conclusions:**

The perioperative ERAS program was associated with faster recovery in patients undergoing radical gastrectomy. For patients with higher BMI (BMI ≥ 28 kg/m^2^), the use of the perioperative ERAS program was more advantageous.

## Introduction

Enhanced recovery after surgery (ERAS) is a series of evidence-based health management strategies in the perioperative period, which reduces psychological and physical traumatic stress responses, postoperative complications, hospital stay times, risks of readmission, incidences of death, and medical costs^1^. Over the years, various ERAS protocols have been developed. Subsequently, the ERAS Society released guidelines for ERAS implementation in different surgical disciplines. ERAS guidelines are based on the highest quality evidence available and as such require updating on a regular basis^2^. To date, the theory of ERAS has been widely used in surgery^2–9^. It was first proposed by the Danish surgeon, Henrik Kehlet^10^, and is now used in the majority of medical institutions in China. Evidence supporting the benefits of ERAS has been increasing rapidly in recent years. In particular, ERAS is valuable in the perioperative period of gastrointestinal tumors, suggesting its clinical significance in gastrointestinal surgery^11^.

China is one of the countries with the highest incidence of gastric cancer. Surgery is currently the most effective approach in increasing the long-term survival of gastric cancer patients. Some studies showed ambiguous outcomes between obese and non-obese patients after gastric cancer surgery^12^. The body mass index (BMI) has been widely used to describe the level of obesity^12^. Current evidence shows that the prevalence of obesity is more common in North China^13^. Moreover, the true impact of obesity on postoperative complications and on long-term survival of patients with gastric cancer is unknown^14^. The purpose of this study was therefore to prospectively investigate the influences of ERAS on postoperative recovery and complications of patients undergoing gastric cancer surgery, and to evaluate the outcomes among patients with different BMI levels at our center.

## Materials and methods

### Patients and randomization

This prospective study was conducted in the advanced ward of Tianjin Medical University Cancer Institute & Hospital from May 2016 to March 2018. Only those patients undergoing radical resection of gastric cancer in the same treatment group were enrolled in the study. After obtaining written consent from all patients, we randomly assigned them into the ERAS (intervention) and non-ERAS (control) groups using a completely randomized design with a 1:1 allocation (gender, age, and TNM stage were used for the allocation). The Central Randomization System (CRS) was used by our staff to screen and randomize the patients. When participants were enrolled, after entering the screen number and the individual’s information, the computer-based randomized number was determined using the Internet, and the allocated group was retrieved on the website of the CRS. The investigators who were responsible for enrollment and intervention, and the responsible physicians, did not participate in the randomization process.

### Inclusion criteria

Inclusion criteria were as follows: (1) patients who received radical gastrectomy; (2) ages of 20–75 years, without gender limits; (2) patients who had complete clinical data; (3) patients who objectively described their symptoms and actively cooperated; and (4) written informed consent from each patient.

### Exclusion criteria

Exclusion criteria were as follows: (1) refusal to sign the consent form; (2) pregnancy or lactating female patients; (3) known allergy to propofol, desflurane, or any other anesthetic agent; (4) patients who received upper abdominal surgery; (5) patients with a severe mental disorder; (6) patients with a history of previous neoadjuvant chemotherapy, radiotherapy, or clinical trial treatment within 3 months; (7) patients who actively participated in another trial where the primary endpoint follow-up was ongoing; (8) patients with complications (bleeding, perforation, or obstruction) caused by gastric cancer; (9) patients with other major medical illnesses of the cardiovascular, respiratory, or immune systems; and (10) patients with severe liver and renal dysfunctions (Child-Pugh ≥ 10; creatinine clearance < 25 mL/min).

### Interventions

During the perioperative period, the 40 patients in the ERAS group were managed in accordance with the ERAS protocol that we optimized^15^, which included preoperative counseling and education about the ERAS program, no smoking or drinking, no bowel preparation, a normal diet until 6 h before surgery, liquid intake until 2 h before surgery, preoperative carbohydrate loading before surgery (100 g glucose/1,000 mL water taken orally at 10 PM on the evening before the surgery and 50 g glucose/500 mL water taken 2–3 h preoperatively). Anesthesia consisted of a combination of epidural analgesia and general anesthesia. To prevent hypothermia, a blanket warming system and warming set for intravenous infusions were used. To prevent postoperative pain, a continuous thoracic epidural infusion of analgesics was given until 2 days after surgery. A non-steroidal anti-inflammatory drug was regularly used to prevent wound pain. Patients started progressive oral feeding and goal-oriented ambulation in postoperative days 1–4. Patients measured the distance of ambulation by the marker in the ward and reported it to the study nurse. If the urinary catheter was not obstructed, it was removed after completion of bladder training. Patients were then encouraged to continue and prolong out-of-bed activities. The remaining 40 patients (non-ERAS group) were intervened by conventional perioperative management as controls. The general interventions in both groups are listed in **Table 1**.

**Table 1:** Perioperative protocols using the enhanced recovery after surgery protocol and conventional treatment

Project	ERAS group	Non-ERAS group
Perioperative projects		
Nutrition assessment (PG-SGA score)	Yes, selective nutritional intervention	No
Preoperative health education	Yes, chief nurse	No
Training of cardiopulmonary function	Yes	No
Prophylactic antithrombotic treatment	Yes, highest Caprini score	Yes, highest Caprini score
Gastrointestinal decompression tube	No	Yes
All-purpose food	Yes	No, fasting
Preoperative bowel preparation	No	Yes
Prophylactic use of antibiotics	Yes	Yes
Intraoperative projects		
Maintain anesthesia under EEG dual-spectrum monitoring	Yes	Yes
Intraoperative ventilation with low tidal volume	Yes	Yes
Transverse abdominal muscle plane block	Yes	No
Ropivacaine infiltration of surgical incisions	Yes	No
Indwelling abdominal cavity drainage tube	If needed	Necessary
Intraoperative body temperature condition monitoring	Yes	Yes
Intraoperative fluid management	Yes	Yes
Postoperative projects		
Early activity	Yes	No
Chewing gum on day 1 after operation	Yes	No
Early removal stomach tube (postoperative 1st day tube)	Yes	No
Early extirpation of abdominal leading tube (placing in operation, extirpation in 3 days after operation)	Yes	No
Early feeding	Yes	No
Postoperative rewarming	Yes	Yes
Preventing nausea and emesis	Yes	Yes
Postoperative respiratory management	Yes	Yes
Active analgesia management	Yes	Yes
Preventing constipation	Yes	No
Early removal catheter	Yes	No
Wound management	Yes	No
Prophylactic antithrombotic treatment	Yes, highest Caprini score	Yes, highest Caprini score
Postoperative health education	Yes	No

### Discharge criteria

Discharge was recommended when the patient met the following criteria: (1) restoring a semi-liquid diet or oral supplementary nutrition; (2) no need for intravenous fluid therapy; (3) no pain or the pain could be controlled with oral analgesics; (4) normal body temperature in the previous 24 h; (5) the patient could freely perform out-of-bed activities; (6) no wound problems; and (7) the patient agreed to be discharged^16^.

### Subgroup analysis

Subgroup analysis was assigned based on the BMI level. China has recommended that BMI ≥ 28 kg/m^2^ is considered as obesity. Moreover, our study purpose was to evaluate the efficacy of ERAS between obese and non-obese patients undergoing gastric cancer surgery, so we set 28 kg/m^2^ as the cut-off value for subgroup analysis. The 40 patients in the ERAS group were divided into 2 subgroups according to their BMI levels, namely group A (BMI < 28 kg/m^2^, *n* = 16) and group B (BMI ≥ 28 kg/m^2^, *n* = 24). Patients in the non-ERAS group were divided in the same way, involving group C (BMI < 28 kg/m^2^, *n* = 18) and group D (BMI ≥ 28 kg/m^2^, *n* = 22), respectively.

### Visual Analogue Scale (VAS) score

The VAS is commonly used to measure panic, depression, fatigue, and pain^17^, and is usually 100 mm in length with anchor descriptors such as “no pain” and “worst pain imaginable.” In the present study, the VAS was reported in centimeters, i.e., on the scale of 0–10 to evaluate postoperative pain. The evaluation was conducted 12 h after surgery.

### Statistical analysis

Statistical analysis was performed using SPSS statistical software for Windows, version 23.0 (SPSS, Chicago, IL, USA). Measurement data were compared between groups using Pearson’s chi-squared test or Fisher’s exact test. Enumeration data were compared between groups using the independent Student’s *t*-test. The normality of data was analyzed using the Kolmogorov-Smirnov test. Statistical significance was set at *P* < 0.05.

## Results

### Baseline characteristics

A total of 80 eligible patients were recruited. The ERAS and control groups comprised 25 and 26 males (*P* = 0.816), respectively. There was no significant difference in TNM stages (I/II/III/IV,7/13/20/0 *vs*. 8/13/19/0, *P* = 0.955) and type of surgery (distal gastrectomy/total gastrectomy/proximal gastrectomy, 30/7/3 *vs*. 29/30/2, *P* = 0.792) on enrollment. Smoking (*P* = 0.502) and drinking (*P* = 0.499) were similar between groups. The mean BMI of the ERAS and non-ERAS groups were 30.7 kg/m^2^ and 30.6 kg/m^2^, respectively (*P* = 0.651). Baseline characteristics of recruited patients are shown in **Table 2**.

**Table 2:** Baseline characteristics of patients in the enhanced recovery after surgery (ERAS) and non-ERAS groups

Clinicopathological variables		Group (*n* = 40)		*P*
		ERAS	Non-ERAS	
Gender	Male	25	26	0.816
	Female	15	14	
Age (years)	Mean ± SD	59 ± 7	60 ± 6	0.582
	≤ 60	19	20	
	> 60	21	20	
TNM stages	I	7	8	0.955
	II	13	13	
	III	20	19
	IV	0	0	
Smoking	Yes	22	19	0.502
	No	18	21	
Drinking	Yes	24	21	0.499
	No	16	19	
Type of surgery	Distal gastrectomy	30	29	0.792
	Total gastrectomy	7	9	
	Proximal gastrectomy	3	2	
BMI (kg/m^2^)	Mean ± SD	30.7 ± 1.7	30.6 ± 1.7	0.651
	< 28	16	18	
	≥ 28	24	22	

### Postoperative recovery of patients in the ERAS and non-ERAS groups

We analyzed the differences in postoperative recoveries between the ERAS and non-ERAS groups. After determining the normality of the data (*P* > 0.05), the Student’s *t*-test was used to compare the data. Patients in the ERAS group had a shorter length of stay, faster postoperative exhaustion and defecation, faster recovery of leukocyte counts, shorter duration of intravenous nutrition, and lower VAS score on the postoperative first day, when compared with those in the non-ERAS group (all, *P* < 0.05); only the time to stopping decline of albumin had no significant difference between the groups (*P* = 0.346). The results showed that use of ERAS during the perioperative period significantly triggered postoperative recovery of patients undergoing gastric cancer surgery (**Table 3**).

**Table 3:** Influence of the enhanced recovery after surgery protocol on postoperative recovery of patients undergoing gastric cancer surgery

	ERAS group	Non-ERAS group	*t*	*P*
Length of stay	7.85 ± 0.921	12.08 ± 1.141	−18.221	<0.001
The time to first postoperative exhaustion	3.53 ± 0.506	4.25 ± 0.588	−5.910	0.015
The time to first postoperative defecation	4.50 ± 0.599	4.680 ± 0.555	−1.873	0.067
The time to return leukocyte count to normal	4.30 ± 0.516	7.40 ± 1.057	−16.662	<0.001
The time to stop decline of albumin	3.55 ± 0.504	3.60 ± 0.496	−1.452	0.346
The time to stop intravenous nutrition	5.05 ± 0.211	7.28 ± 0.452	−27.966	<0.001
VAS score on the postoperative first day	3.08 ± 0.572	4.55 ± 0.504	−12.234	<0.001

### Subgroup analyses of the BMI

Patients were allocated into 4 groups depending on their BMI levels; in the ERAS group: group A (BMI < 28 kg/m^2^, *n* = 16), group B (BMI ≥ 28 kg/m^2^, *n* = 24), and in the non-ERAS group: group C (BMI < 28 kg/m^2^, *n* = 18), group D (BMI ≥ 28 kg/m^2^, *n* = 22). We analyzed the 4 groups in pairs, with the results summarized in **Tables 4 and 5**. Compared with the patients in group C, perioperative intervention of ERAS in patients whose BMI < 28 kg/m^2^ (group A) achieved better postoperative recovery, showed a shorter length of stay, faster postoperative exhaustion and defecation, faster recovery of leukocyte counts, shorter duration of intravenous nutrition, and a lower VAS score on the postoperative first day (all, *P* < 0.05).

**Table 4:** Comparison of the 4 subgroups after allocation based on the body mass index level

	Group A	Group B	Group C	Group D
Length of stay	7.45 ± 0.510	8.25 ± 1.070	11.85 ± 1.348	12.30 ± 0.865
The time to first postoperative exhaustion	3.40 ± 0.503	3.65 ± 0.489	4.25 ± 0.716	4.25 ± 0.444
The time to first postoperative defecation	4.30 ± 0.571	4.70 ± 0.571	4.95 ± 0.605	5.05 ± 0.510
The time to return leukocyte count to normal	4.40 ± 0.503	4.20 ± 0.523	6.55 ± 0.510	8.25 ± 0.716
The time to stop decline of albumin	3.60 ± 0.503	3.50 ± 0.513	4.65 ± 0.489	4.55 ± 0.510
The time to stop intravenous nutrition	5.00 ± 0.001	5.10 ± 0.308	7.00 ± 0.001	7.55 ± 0.510
VAS score on the postoperative first day	3.35 ± 0.489	2.80 ± 0.523	4.40 ± 0.503	4.70 ± 0.470

**Table 5:** Comparison of the 4 subgroups after allocation based on the body mass index

	Group A *vs.* Group C		Group B *vs.* Group D		Group A *vs.* Group B		Group C *vs.* Group D	
	*t*	*P*	*t*	*P*	*t*	*P*	*t*	*P*
Length of stay	−13.647	<0.001	−13.167	<0.001	−3.018	0.005	−1.256	0.217
The time to first postoperative exhaustion	−4.344	<0.001	−4.06	<0.001	−1.594	0.119	−0.987	0.543
The time to first postoperative defecation	−3.494	<0.001	−2.043	0.048	−2.214	0.033	−0.565	0.575
The time to return leukocyte count to normal	−13.422	<0.001	−20.419	<0.001	1.233	0.225	−8.643	<0.001
The time to stop decline of albumin	−6.694	<0.001	−6.489	<0.001	0.623	0.537	0.632	0.531
The time to stop intravenous nutrition	−3.21	<0.001	−18.383	<0.001	−1.453	0.154	−4.819	<0.001
VAS score on the postoperative first day	−6.694	<0.001	−12.08	<0.001	3.434	0.001	−1.949	0.059

Compared with patients in group D, perioperative intervention of ERAS in patients whose BMI < 28 kg/m^2^ (group B) showed better postoperative recovery, a shorter length of stay, faster postoperative exhaustion and defecation, faster recovery of leukocyte counts, shorter duration of intravenous nutrition, and lower VAS score on the postoperative first day (all, *P* < 0.05).

The comparison of group A *vs.* group B showed that group A had a shorter length of stay and faster postoperative defecation and lower VAS score on the postoperative first day than those of group B. In a similar manner, patients in the non-ERAS group with BMI < 28 kg/m^2^ (group C) showed faster recovery of leukocyte counts and shorter duration of intravenous nutrition than those of group D (*P* < 0.05).

### Postoperative complications

There were 13 cases of intermuscular deep vein thrombosis of the lower extremities, 1 case in group A, 2 cases in both groups B and C, and 8 cases in group D. Two patients developed postoperative lung infections in group D. The incidence of intermuscular deep vein thrombosis of lower extremities was significantly higher in group D than in group B (χ^2^ = 4.800, *P* = 0.028), suggesting that perioperative intervention of ERAS in patients with high BMI (BMI ≥ 28 kg/m^2^) significantly decreased the incidence of intermuscular deep vein thrombosis of the lower extremities. A higher incidence of postoperative intermuscular deep vein thrombosis of lower extremities and lung infection were found in group D, when compared with those of other groups. Other complications such intestinal obstruction, leakage, and anastomosis failure were not observed in any groups (**Table 6**), indicating that implementation of ERAS in patients with high BMI had advantages in reducing postoperative complications.

**Table 6:** Complications in patients undergoing gastric cancer surgery who were intervened by the enhanced recovery after surgery protocol and the body mass index

	Group A	Group B	Group C	Group D
Intermuscular deep vein thrombosis of lower extremities (*n*)	1	2	2	8
Lung infection (*n*)	0	0	0	2
Intestinal obstruction (*n*)	0	0	0	0
Leakage (*n*)	0	0	0	0
Anastomosis failure (*n*)	0	0	0	0

## Discussion

ERAS is a surgical concept that was updated from conventional health management. It aims to reduce the psychological burden, physical stress of patients, and accelerate their postoperative rehabilitation. However, the feasibility and safety of ERAS has been questioned because of the large differences between ERAS and conventional approaches, and as a result, the use of ERAS was restricted^18^. In recent years, clinical outcomes of ERAS have been gradually recognized and popularized in multiple clinical fields. Although the use of ERAS in gastric cancer surgery started late, it has been developing rapidly. Increasing data have shown the safety of ERAS during the perioperative period of gastric cancer surgery, and ERAS has achieved more advantages in rapid recovery when compared with those of conventional care.

In the present study, postoperative recovery variables were significantly better in the ERAS group than those in the non-ERAS group, including the time to first postoperative exhaustion, postoperative defecation, returning leukocyte counts to normal, halting decline of albumin, stopping intravenous nutrition, and VAS scores at the postoperative first day, which is consistent with a previous study^19^. The speed of the first postoperative exhaustion and defecation reflects the recovery of intestinal motility after gastric surgery. The recovery of leukocyte counts indicates the postoperative state of infections. Postoperative nutrition is acceptable until albumin is no longer declined and intravenous nutrition is no longer needed. In addition, the postoperative VAS score directly indicates the quality of life of patients during the postoperative recovery period. Collectively, the results of this study confirmed that ERAS accelerated the recovery of intestinal motility, control of infection, nutrition loss, and postoperative pain in patients undergoing gastric cancer surgery.

On different levels, recent evidence has confirmed the influence of obesity on the short-term efficacy and the prognosis of gastric cancer surgery^20,21^. For example, for patients undergoing curative gastric cancer surgery, those who were overweight or mildly-to-moderately obese (BMI 23 < 30 kg/m^2^) preoperatively had better overall survival and disease-specific survival than normal-weight patients^20^. Another study reported that compared with the BMI, a body shape index was an independent risk factor for overall complications in patients with gastric cancer^21^. In the present study, patients were categorized into 4 subgroups based on the BMI, and we determined the influence of ERAS on postoperative recovery of obese patients undergoing gastric cancer surgery. The results showed that patients with a high BMI recovered more slowly than those with a normal range of BMI, when treated with conventional care. Moreover, patients with high BMI who were treated with ERAS presented better outcomes in the time to first postoperative exhaustion, postoperative defecation, returning leukocyte counts to normal, stopping decline of albumin, and stopping intravenous nutrition, than those not following the ERAS protocol. Notably, the incidence of lung infection and deep vein thrombosis of the lower extremities were significantly lower in patients with high BMI who followed the ERAS protocol. As previously mentioned in the Introduction, the prevalence of obesity is significantly higher in North China, therefore ERAS is more meaningful in this region.

Our study had some limitations, such as the single center nature of this study and the limited number of patients. Every recruited patient was strictly selected according to the inclusion and exclusion criteria, which caused a decrease in the number of eligible patients. In addition, as in all clinical randomized trials investigating ERAS, this study was not blinded. The study coordinator and the caregivers all had to know the treatment arm in order to ensure adherence to the protocol. This might have resulted in some level of performance and detection bias. Thus, our findings should be further studied in the clinical settings of other hospitals.

## Conclusions

This study showed that patients undergoing radical gastrectomy for gastric cancer had a faster recovery after perioperative treatment using the ERAS protocol. For patients with a high BMI (BMI ≥ 28 kg/m^2^), the benefit was more obvious after receiving the ERAS protocol during the perioperative period.
